# Advanced glycation end-products regulate extracellular matrix-adipocyte metabolic crosstalk in diabetes

**DOI:** 10.1038/s41598-019-56242-z

**Published:** 2019-12-24

**Authors:** Clarissa Strieder-Barboza, Nicki A. Baker, Carmen G. Flesher, Monita Karmakar, Christopher K. Neeley, Dominic Polsinelli, Justin B. Dimick, Jonathan F. Finks, Amir A. Ghaferi, Oliver A. Varban, Carey N. Lumeng, Robert W. O’Rourke

**Affiliations:** 1 Department of Surgery, University of Michigan Medical School, Ann Arbor, MI USA; 2 Department of Pediatrics and Communicable Diseases, University of Michigan Medical School, Ann Arbor, MI USA; 3 Graduate Program in Immunology, University of Michigan Medical School, Ann Arbor, MI USA; 4 Graduate Program in Cellular and Molecular Biology, University of Michigan Medical School, Ann Arbor, MI USA; 5 Undergraduate Research Opportunity Program, University of Michigan, Ann Arbor, MI USA; 6 Department of Surgery, Ann Arbor Veterans Affairs Healthcare System, Ann Arbor, MI USA

**Keywords:** Mechanisms of disease, Type 2 diabetes, Metabolic disorders, Obesity

## Abstract

The adipose tissue extracellular matrix (ECM) regulates adipocyte cellular metabolism and is altered in obesity and type 2 diabetes, but mechanisms underlying ECM-adipocyte metabolic crosstalk are poorly defined. Advanced glycation end-product (AGE) formation is increased in diabetes. AGE alter tissue function via direct effects on ECM and by binding scavenger receptors on multiple cell types and signaling through Rho GTPases. Our goal was to determine the role and underlying mechanisms of AGE in regulating human ECM-adipocyte metabolic crosstalk. Visceral adipocytes from diabetic and non-diabetic humans with obesity were studied in 2D and 3D-ECM culture systems. AGE is increased in adipose tissue from diabetic compared to non-diabetic subjects. Glycated collagen 1 and AGE-modified ECM regulate adipocyte glucose uptake and expression of AGE scavenger receptors and Rho signaling mediators, including the *DIAPH1* gene, which encodes the human Diaphanous 1 protein (hDia1). Notably, inhibition of hDia1, but not scavenger receptors RAGE or CD36, attenuated AGE-ECM inhibition of adipocyte glucose uptake. These data demonstrate that AGE-modification of ECM contributes to adipocyte insulin resistance in human diabetes, and implicate hDia1 as a potential mediator of AGE-ECM-adipocyte metabolic crosstalk.

## Introduction

Adipose tissue metabolic dysfunction underlies the pathogenesis of obesity-associated metabolic disease, including type 2 diabetes (DM), and alterations in the adipose tissue extracellular matrix (ECM) have been implicated^1–5^. Using a novel human ECM-adipocyte co-culture system, we recently demonstrated that adipose tissue ECM regulates adipocyte cellular metabolism in a disease-specific manner, with rescue of cellular insulin resistance in DM adipocytes by non-diabetic (NDM) ECM^6^. Nonetheless, mechanisms underlying ECM-adipocyte crosstalk and the cascade of events that lead to adipocyte metabolic dysfunction in DM are poorly defined.

Advanced glycation end-products (AGE), the products of nonenzymatic glycation and oxidation of proteins and lipids,^7^ are a putative contributor to cellular dysfunction in response to hyperglycemia associated with DM. AGE disrupt protein tertiary structure and alter a wide range of protein functions and structural interactions. AGE also act as ligands for scavenger receptors expressed by many cell types, including Receptor for Advanced Glycation End-products (RAGE) and CD36, triggering multiple signaling pathways with diverse effects on cell function^8,9^. AGE are increased in multiple tissues in DM and contribute to end-organ disease^7,10–14^, but their role in adipose tissue is not well-described. AGE reduce adipogenic differentiation in human mesenchymal cells^15^ and impair insulin sensitivity in 3T3-L1 adipocytes cultured in high-glucose-medium^16^, suggesting their involvement in adipose tissue insulin resistance. AGE represent a putative mechanism underlying ECM-adipocyte interactions, possibly via signaling through Rho GTPases, a downstream signaling pathway activated by AGE^17^. Rho GTPases regulate insulin-stimulated glucose uptake in muscle and adipose tissue^18^, and AGE activate Rho signaling in endothelial and microglial cells^19–23^, but no published data link AGE and Rho signaling in adipose tissue dysfunction.

The present study evaluates mechanisms by which AGE regulate ECM-adipocyte metabolic crosstalk in human adipose tissue. We hypothesized that AGE-modified ECM impairs adipocyte glucose metabolism via AGE-receptors and Rho signaling. Manipulation of the adipocyte microenvironment was accomplished with high glucose culture of recombinant collagen and ECM. Antagonist antibodies to AGE-receptors and a Rho signaling mediator small molecule inhibitor were used to identify signaling pathways underlying AGE-mediated ECM-adipocyte crosstalk. A 3D human ECM-adipocyte co-culture system was used to simulate the adipose tissue environment *in vitro*. This system provides advantages over other 3D culture methods that use hydrogels or collagens in that it is derived from native adipose tissue and thus provides a more physiologic environment for adipocyte culture.

## Results

### AGE are increased in adipose tissue of obese diabetic patients

**Figure 1:**
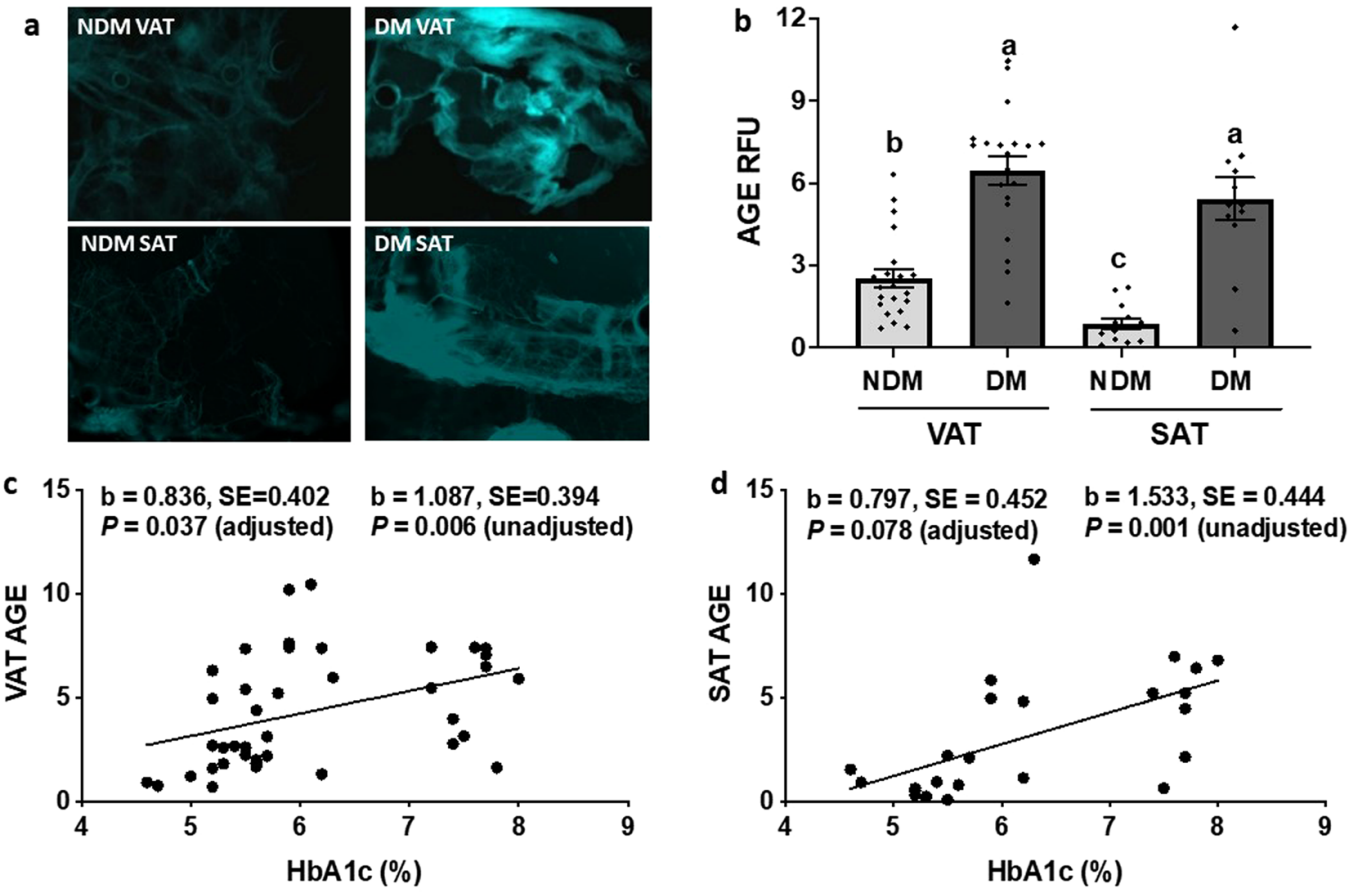
AGE are increased in diabetic human adipose tissue. (**a**) Representative fluorescence microscopy images and (**b**) quantified mean relative fluorescence intensity units (RFU) of AGE expression in human DM and NDM VAT and SAT. Bars with different letters indicate *P* < 0.050 in multiple comparison analysis. (**c**,**d**) Association of VAT or SAT AGE fluorescence intensity and serum HbA1c percentage for entire cohort. Linear model b value (**b**), standard error (SE) and P-values shown are unadjusted and adjusted for age and sex. VAT: n = 21 NDM, 19 DM subjects; SAT: n = 13 NDM, 12 DM subjects.

To determine if AGE are increased in adipose tissue in human obesity and DM, we measured AGE levels in subcutaneous (SAT) and visceral (VAT) adipose tissues from obese DM and NDM patients using fluorescence microscopy^24,25^. AGE levels were higher in DM compared to NDM tissues and similar between SAT and VAT (Fig. 1A,B). Analysis of linear mixed models revealed that VAT and SAT AGE levels correlated directly with percentage of glycated hemoglobin (HbA1c; Fig. 1C,D). No differences were observed in plasma AGE levels between obese DM and NDM subjects (P = 0.818).

### Glycated collagen 1 regulates glucose metabolism and Rho-AGE receptor gene expression in adipocytes in 2D culture

**Figure 2:**
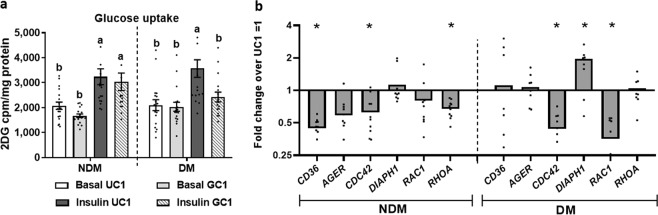
Glycated collagen 1 regulates adipocyte insulin signaling and Rho and AGE scavenger receptor expression. Preadipocytes from human VAT were differentiated into mature adipocytes in 2D culture +/− unglycated or glycated recombinant human collagen 1 (UC1, GC1), then studied with glucose uptake assay or RT-qPCR. (**a**) Glucose uptake in non-stimulated (basal) or insulin-stimulated conditions. Ordinate: mean glucose uptake measured by ^3^H-2D-glucose in cell lysates (cpm) normalized to cell lysate protein concentration (mg/ml); bars with different letters indicate P < 0.050; n = 15 NDM, 15 DM subjects. (**b**) Gene expression studied with RT-qPCR. Ordinate: mean fold difference in transcript level in GC1 arm relative to UC1 arm referent = 1; *P < 0.050, comparing transcript levels in GC1 arm vs. UC1 arm; n = 12 NDM, 12 DM subjects.

We next explored the effects of glycated collagen 1 (GC1) as a surrogate for AGE on adipocyte glucose metabolism and gene expression in 2D culture. Given its stronger association with metabolic disease, for these and all subsequent experiments, we studied adipocytes from VAT. GC1, relative to UCI, had no effect on basal glucose uptake, but decreased insulin-stimulated glucose uptake in DM but not NDM adipocytes (Fig. 2A). These results suggest that glycated collagen 1 interferes with adipocyte insulin signaling and insulin-stimulated glucose uptake, with the latter effect restricted to DM adipocytes.

Using real-time quantitative polymerase chain reaction (RT-qPCR), we next evaluated the effects of GC1 on gene expression of *AGER* (Advanced Glycosylation End-Product Specific Receptor, gene designation for RAGE)*, CD36*, and Rho signaling mediators *CDC42* (Cell Division Cycle 42), *DIAPH1* (Rho-dependent Diaphanous Related Formin 1, gene designation for hDia1), *RAC1* (Rac Family Small GTPase 1) and *RHO*A (Ras Homolog Family Member A) in VAT adipocytes from obese DM and NDM subjects. In NDM patients, GC1 decreased expression of *CD36*, *CDC42*, and *RHOA* in NDM compared with UC1 adipocytes, whereas in DM adipocytes, GC1 decreased the expression of *CDC42* and *RAC1* and increased expression of *DIAPH1* (Fig. 2B). Together, these data suggest that glycated collagen 1 differentially regulates expression of AGE-receptors and Rho signaling mediators in adipocytes from obese DM and NDM subjects, and induces *DIAPH1* expression in a DM-specific manner.

### AGE-modified ECM impairs adipocyte glucose uptake in 3D culture

**Figure 3:**
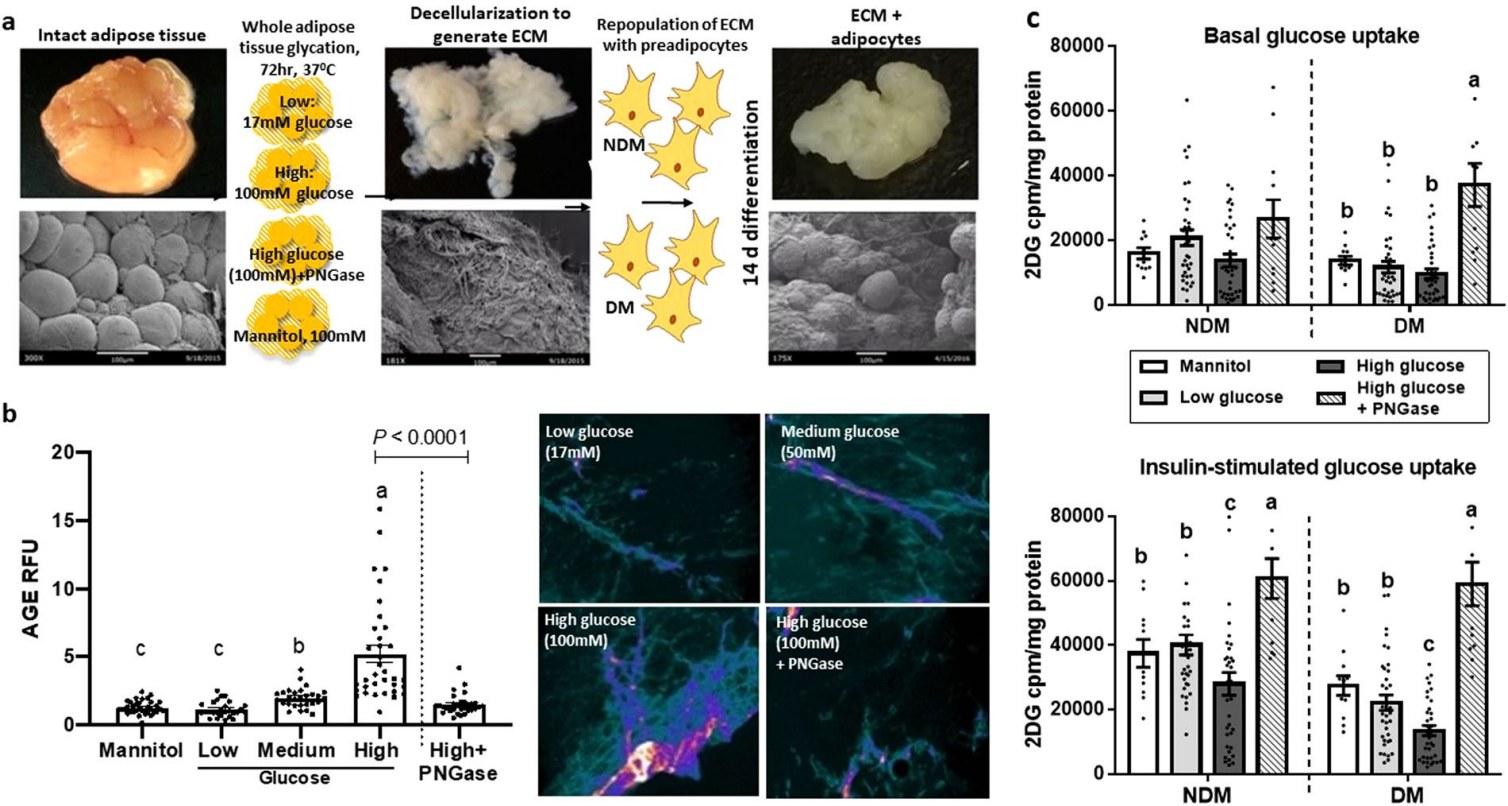
AGE-modified adipose tissue ECM regulates adipocyte cellular metabolism. (**a**) Strategy for creation of ECM-adipocyte cultures, including macroscopic photographs and scanning electron micrographs. (**b**) Quantified mean fluorescence of VAT treated with indicated glucose or mannitol concentrations for 72 h, with or without PNGase for final 24 h of treatment. Bars with different letters indicate P < 0.050. (**c**) Basal and insulin-stimulated glucose uptake in preadipocytes from visceral adipose tissue of DM and NDM obese subjects differentiated into mature adipocytes in disease-matched (DM or NDM) ECM prepared from tissues treated with 17 mM (Low) or 100 mM (High) glucose or 100 mM mannitol, +/− PNGase. Ordinates: mean glucose uptake measured by ^3^H-2D-glucose in cell lysates (cpm) normalized to cell lysate protein concentration (mg/ml). Bars with different letters indicate P < 0.050; n = 29 NDM, 25 DM subjects.

We next studied the effects of AGE-modified ECM on adipocyte metabolism in a 3D-ECM-adipocyte culture system previously described by our laboratory^6^, targeting AGE-modification to the ECM by treating adipose tissue with high glucose concentrations prior to ECM isolation (Fig. 3A). We first optimized AGE-induction on ECM using high glucose culture by treating adipose tissues for 72 hours with medium containing 17 mM, 50 mM, or 100 mM glucose, or 100 mM mannitol (negative control), prior to isolation of ECM. We also studied ECM isolated from tissues treated with the deglycosylating enzyme PNGase for the final 24 hours of the 72-hour glucose conditioning. Fluorescence microscopy analysis revealed that 100 mM glucose treatment induced AGE on ECM to levels approximating those observed in native DM VAT (Fig. 3B, compare with Fig. 1B), while PNGase markedly decreased AGE levels (b = −0.45 ± 0.12; p < 0.001). There was a significant interaction between the PNGase and glycation effect, such that the decrease in the AGE levels on addition of PNGase was significantly higher for the adipocytes in high glucose conditions compared to the decrease observed in low glucose conditions (b = −1.14 ± 0.20; p < 0.001). Based on these results, for all subsequent experiments, we used high glucose (100 mM) treatment to designate AGE-modified ECM and low glucose (17 mM), mannitol (100 mM), and high glucose followed by PNGase treatment as controls.

We next evaluated glucose uptake in AGE-modified 3D-ECM-adipocyte culture. ECM isolated from treated VAT was seeded with VAT preadipocytes, combining NDM ECM with NDM preadipocytes, or DM ECM with DM preadipocytes, thus recapitulating diseased (DM) and non-diseased (NDM) tissues. DM ECM-adipocyte cultures treated with low or high glucose manifested decreased basal and insulin-stimulated glucose uptake compared with NDM cultures, confirming the DM-specific defect in glucose uptake previously observed by our laboratory with this 3D ECM-adipocyte system^6^. ECM prepared from tissues treated with 100 mM mannitol, in contrast, had no effect on glucose uptake in either NDM or DM ECM-adipocyte cultures. High glucose AGE-modified ECM significantly decreased insulin-stimulated glucose uptake relative to low glucose-conditioned ECM in both NDM and DM ECM-adipocyte cultures but did not affect basal glucose uptake, even after controlling for diabetes status, age and sex. Finally, PNGase treatment of high glucose-treated ECM abrogated its inhibitory effect on insulin-stimulated glucose uptake in NDM and DM ECM-adipocyte cultures after controlling for age and sex (Fig. 3C). Together these data demonstrate that AGE-modified ECM attenuates adipocyte insulin-stimulated glucose uptake, with this effect being more pronounced in DM ECM-adipocyte cultures.

### AGE-ECM impairment of adipocyte glucose uptake is hDia1-dependent

**Figure 4:**
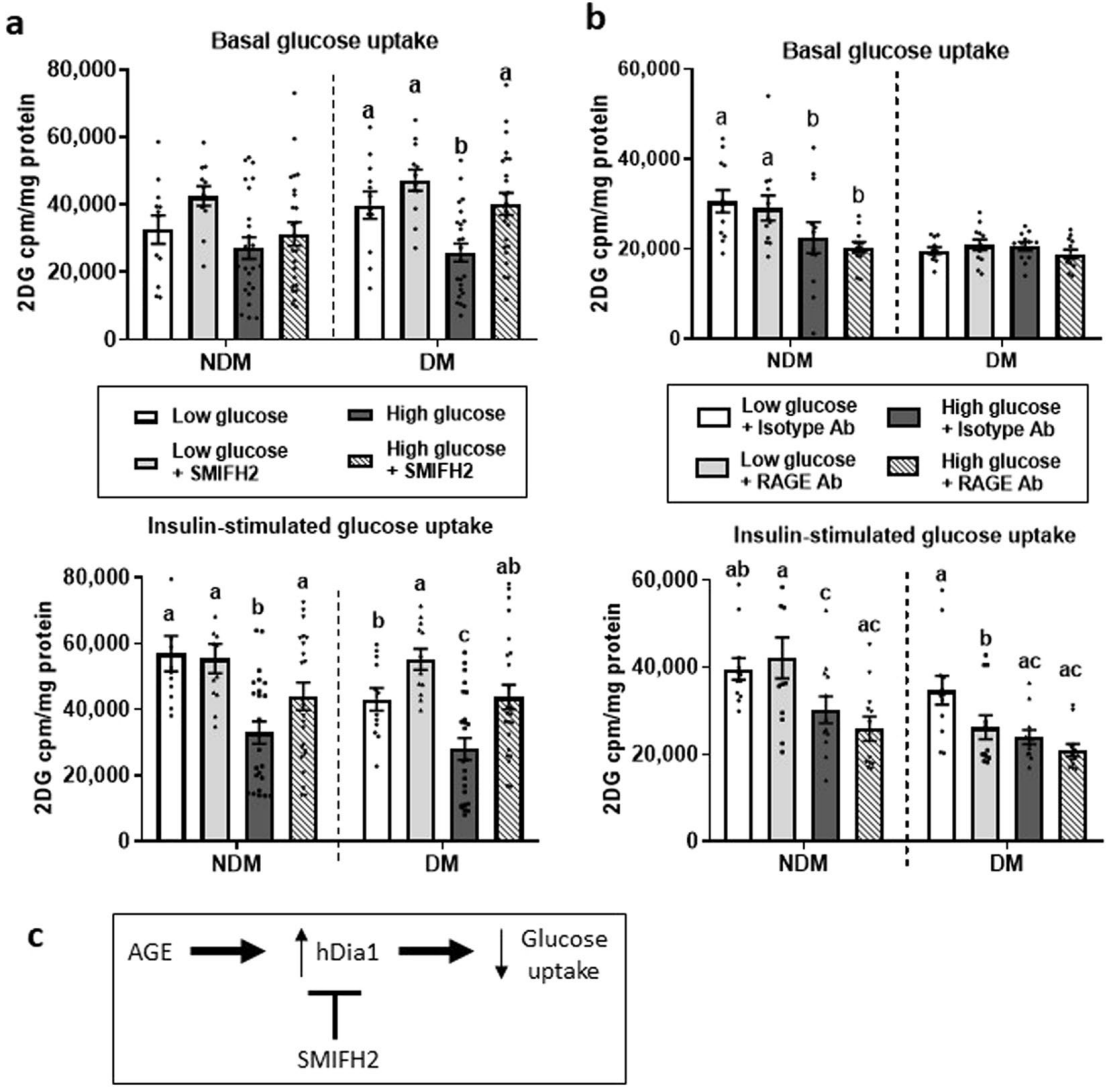
hDia1 regulates AGE-mediated ECM-adipocyte metabolic crosstalk in 3D-ECM culture. Preadipocytes were differentiated *in vitro* into mature adipocytes +/− (**a**) the hDia1 small molecule inhibitor SMIFH2 or (**b**) AGE receptor (RAGE) antagonist or isotype control antibodies in disease-matched (NDM or DM) ECM prepared from visceral adipose tissue treated with Low (17 mM) or High (100 mM) glucose, then studied with glucose uptake assay without (basal) or with insulin stimulation. Ordinates: mean ^3^H-2D-glucose uptake (cpm) normalized to cell lysate protein concentration (mg/ml). Bars with different letters indicate P < 0.050; n = 22 NDM,16 DM subjects. (**c**) A simplified model for AGE regulation of adipocyte glucose uptake.

We next determined the roles of hDia1, RAGE, and CD36 in regulating ECM-adipocyte crosstalk by evaluating glucose uptake in ECM-adipocyte cultures matched by diabetic status treated with antibody directed towards RAGE or CD36, or a small molecule formin homology 2 domain inhibitor (SMIFH2), which inhibits hDia1 function (**refs**). SMIFH2 attenuated AGE-ECM-mediated impairment of insulin sensitivity in NDM and DM ECM-adipocyte cultures as observed by increased glucose uptake (Fig. 4A). These effect was similarly observed in basal glucose uptake in DM but not in NDM ECM-adipocyte cultures. The improvement of glucose uptake with SMIFH2 was increased in magnitude in high glucose compared to low glucose ECM-adipocyte cultures, and greater in DM compared to NDM cultures. In contrast, RAGE neutralizing antibody, relative to treatment with isotype control antibody, had no effect on glucose uptake in low or high glucose-treated NDM and DM ECM-adipocyte cultures (Fig. 4B). Similarly, CD36 neutralizing antibody, relative to isotype control antibody, had no effect on glucose uptake in low glucose-treated NDM and DM ECM-adipocyte cultures (Supplemental Fig. 1). These observations imply that regulation of adipocyte glucose uptake by AGE-modified ECM is partially mediated by hDia1, effects that are more pronounced in the context of increased AGE levels associated with DM and independent of RAGE or CD36.

## Discussion

The role of AGE in regulating adipose tissue dysfunction in the context of DM remains poorly understood. AGE are increased in kidney, muscle, skin, and liver in obese DM humans^10–12^, and in adipose tissue in obese rodents^13^ but study of AGE in human adipose tissue is sparse. A single report demonstrates increased AGE levels in SAT in obese compared to lean humans^26^, with no published reports of AGE levels in human VAT. We demonstrate that elevated AGE levels in VAT and SAT is a feature of human obesity-associated DM. In contrast, we observed no correlation between plasma AGE levels and obesity or DM. Some prior studies demonstrate decreased plasma AGE levels in obesity^10,26,27^, while others show the opposite^28^. Different patient populations and detection assays may account for these conflicting data. Of note, decreased AGE plasma levels have been associated with increased adipose tissue AGE levels^26^, suggesting that tissues may act as an ‘AGE-sink’, reducing plasma levels. Our data are consistent with these data in that they support that plasma AGE levels do not accurately reflect tissue levels in human metabolic disease.

We demonstrate that in 2D-culture, GC1 attenuates adipocyte glucose uptake in DM adipocytes, consistent with prior studies demonstrating a similar effect of glycated albumin or high glucose in 3T3L1 adipocytes^16,29–31^. We observed distinct effects of GC1 on adipocyte glucose metabolism depending on DM status, suggesting disease-specific effects of AGE-modified collagen on cellular metabolism. The detrimental effects of GC1 on DM but not NDM adipocytes suggest that intrinsic cellular defects associated with metabolic disease render adipocytes susceptible to metabolic impairment by glycated collagen.

To better model the complexity of native adipose tissue, we used a 3D-ECM-adipocyte culture system that permits isolated manipulation of ECM. Using this system, we observed an inhibitory effect of AGE-modified ECM on adipocyte glucose uptake that was reversed with PNGase treatment, suggesting that glycated ECM contributes to the well-established state of adipocyte cellular insulin resistance associated with DM^32–34^. In addition, the effects of AGE-modified ECM on glucose uptake were more profound than those of GC1 in 2D culture, suggesting that glycation of ECM proteins in addition to collagen 1 are required for maximal effects of AGE on adipocyte metabolism, and likely involve multiple AGE moieties on multiple ECM proteins. Notably, the effect of AGE-modified ECM on adipocyte glucose metabolism was greater in DM compared to NDM ECM-adipocyte cultures, and furthermore, PNGase treatment of AGE-modified ECM increased adipocyte glucose uptake to levels higher than in non-AGE modified ECM not treated with PNGase in both NDM and DM cultures. These observations suggest that a degree of pre-existing glycosylation carried over from the *in vivo* environment present on both obese DM and NDM ECM already impairs adipocyte metabolism, that further *in vitro* AGE induction on ECM has additional detrimental effects on adipocyte glucose uptake, and finally, that complete *in vitro* removal of ECM glycosylation improves adipocyte glucose uptake to levels beyond that observed in untreated ECM.

AGE mediate their effects in part by binding scavenger receptors on a wide range of cells and triggering downstream signaling pathways. Dominant among these scavenger receptors is RAGE, activation of which induces cellular and systemic insulin resistance in multiple models^35–37^. RAGE expression in epicardial adipose tissue correlates directly with epicardial fat thickness and indirectly with GLUT4 expression^38^, while RAGE expression in SAT correlates indirectly with coronary artery disease^39^. AGE also signal through the scavenger receptor CD36; the role of CD36 in metabolic disease is controversial, with some data demonstrating beneficial cellular and systemic metabolic effects, while other data demonstrate the opposite^40–42^. We observed no significant effect of GC1 on *AGER* expression, decreased CD36 transcript levels in response to GC1 in NDM adipocytes, and finally, no effect of antibody-inhibition of RAGE or CD36 on glucose uptake in adipocytes cultured in AGE-modified ECM. These observations suggest that AGE-ECM exerts its effects on adipocyte insulin resistance independent of RAGE and CD36; the observed downregulation of CD36 by GC1 in NDM adipocytes suggests that this receptor may mediate cellular responses to AGE distinct from insulin resistance; further research will be necessary to explore this hypothesis.

Data regarding the role of Rho GTPases in regulating cellular and systemic metabolism are conflicting. The Rho signaling family is comprised of many mediators with complex tissue- and context-specific functions, precluding broad generalizations. Rac1, for example, potentiates skeletal muscle glucose uptake via regulation of cytoskeletal mobilization of GLUT4 transporters^43^, but does not appear to play a similar role in adipocytes^44,45^. In contrast, RhoA and ROCK promote insulin resistance in myocytes^46^ and pancreatic beta cells^47^. Other data demonstrate that Rho signaling is upregulated in adipose and other tissues in murine and human obesity^48–51^, and that Rho inhibition improves systemic metabolism^52–54^. Together, these observations suggest both positive and negative effects of Rho signaling on cellular and systemic metabolism. The mammalian Diaphanous-related formin protein family are Rho GTPase effectors that mediate actin polymerization and microtubule stabilization. Human Diaphanous 1 (hDia1) and its murine homolog mDia1, is the best studied of the Dia family, and mediates cell migration via AGE/RAGE signaling in non-adipocyte cell types^55,56^. Few data describe the role of hDia1 in adipocytes, with a single report demonstrating that mDia1 regulates adipogenic differentiation of murine mesenchymal stem cells^57^. We demonstrate increased *DIAPH1* transcript levels in DM but not NDM adipocytes in response to GC1. Furthermore, we observed that treatment with SMIFH2 attenuated AGE-ECM-induced inhibition of insulin-stimulated glucose uptake in NDM and DM ECM-adipocyte cultures, suggesting a role for hDia1 in promoting cellular insulin resistance in adipocytes in response to AGE. These observations, along with prior data linking adipose tissue Rho activity to insulin resistance^48–51^, suggest that DM adipocytes may be more susceptible than NDM adipocytes to increased hDia1 expression in response to AGE, contributing to cellular insulin resistance in DM. Consistent with this hypothesis is the observed increased in *DIAPH1* transcript levels in response to GC1 in DM but not NDM adipocytes.

We studied VAT given its stronger association with metabolic disease, and due to limitations in access to SAT. DM subjects were older, included more men, and had a higher prevalence of other metabolic diseases than NDM subjects; we adjusted post-hoc for age, sex, and BMI in comparisons between NDM and DM groups, and comparisons between AGE-treated and non-AGE-treated arms were matched for each subject as paired analyses, and thus internally controlled. Nonetheless, larger studies will be required to rigorously address the role of age, sex, medication use, and other clinical variables in contributing to DM-specific differences in ECM-adipocyte crosstalk. PNGase F is known to be specific for N-glycans, but our data suggest that it removes AGE moieties as well, at least as measured by fluorescence. It is possible that PNGase F may have broader specificity than currently understood, or alternatively, we may have observed removal of AGE-modified N-glycans by PNGase F. Future detailed studies using mass spectrometry and other biochemical approaches will be required to answer these questions. We studied the effects of glycated collagen on adipocytes by adding soluble glycated collagen directly to the culture media, but study of collagen-coated culture plates may provide different results, which will be addressed in future research. *In vitro* high glucose conditioning is not a perfect model for AGE-induction, likely inducing different profiles of AGE moieties than those present *in vivo*. Nonetheless, this is an accepted method of AGE induction, and in our model system, achieved AGE levels similar to those observed in DM adipose tissues. Future research will study alternative AGE-induction methods, including ribose- or glyoxalate-conditioning^58,59^. Finally, while frequently used as an inhibitor of hDia1^60,61^, SMIFH2 also inhibits other formin-domain-containing proteins, which may regulate AGE effects on adipocyte metabolism. Future experiments studying targeted knockdown of hDia using shRNA will clarify this issue, methodology which to date has not been feasible in 3D-ECM-adipocyte culture.

We demonstrate that AGE mediate ECM-adipocyte metabolic crosstalk in human adipocytes, possibly via regulation of the Rho signaling mediator hDia1. These data implicate AGE-modified adipose tissue ECM and Rho signaling as contributing mechanisms to adipocyte metabolic dysfunction in DM.

## Methods

### Human subjects

All human subjects underwent informed consent for study participation and were enrolled with Institutional Review Board approval at University of Michigan and Ann Arbor Veterans Affairs Healthcare System. Patient enrollment and all methods were performed in accordance with all methods were performed in accordance with all relevant institutional, federal, and international guidelines and regulations. VAT from the greater omentum, SAT from the abdominal wall, and peripheral blood were collected from obese subjects during bariatric surgery (Table 1). Due to limitations in tissue amounts, tissue and cell samples subsets of a total of 165 subjects were used for each experiment, with numbers of subjects for each experiment reported in figure legends. DM subjects were defined by clinical diagnosis requiring medication and hemoglobinA1c (HbA1c) >= 6.5%. Non-diabetic (NDM) subjects were defined by no clinical history of diabetes and HbA1c <5.7% per American Diabetes Association criteria^62^.

**Table 1:** Subject demographics.

	DM (n = 75)	NDM (n = 90)	P-value*
**Clinical characteristics**			
Sex (% male)	61%	51%	0.019
Age (mean, SD, years)	55 (11)	44 (11)	<0.001
BMI (mean, SD, kg/m^2^)	44 (6)	46 (6)	0.346
HbA1c (mean, SD, %)	7.1% (1.0%)(1.0%)	5.4% (0.3%)	<0.001
**Comorbid diseases (%)**			
Sleep apnea	85%	67%	0.383
Hypertension	70%	41%	<0.001
Dyslipidemia	72%	23%	<0.001
**Medications (%)**			
ACE inhibitor	42%	12%	<0.001
β-blocker	29%	12%	0.025
Insulin	42%	0%	<0.001
Metformin	75%	4%	<0.001
Statin	67%	19%	<0.001
Sulfonylurea	20%	0%	<0.001
Thiazolidinedione	4%	0%	0.076
GLP-1 modulator	4%	0%	0.041

*Independent t-test and Fisher’s exact test were used to compare continuous and dichotomous variables respectively between DM and NDM groups; SD: standard deviation.

### Fluorescence microscopy

AGE levels in adipose tissue and plasma were quantified using fluorescence microscopy based on AGE autofluorescence^24,25^. Adipose tissue was frozen in liquid nitrogen, embedded in optimal cutting temperature compound, sectioned (100 μm) on a Microm HM500OM cryostat (GMI Inc., Ramsey, MN, USA), and imaged on an Olympus IX-81 fluorescent microscope using 10X objective, excitation 377 nm +/− 25nm, emission 447 nm +/− 30nm. Four grayscale TIFF images were captured for each slide, 9.6 ms exposure, ISO 200. Pixel intensities were measured with ImageJ software. For plasma, 5 μl of plasma was spotted on a glass slide, dried, then imaged with identical technique except 8.78 ms exposure.

### Adipose tissue ECM isolation

Adipose tissue ECM isolation was performed as described^6^, based on modifications of published protocols^63–65^. VAT explants were freeze-thawed from −80 °C, 20 min to 37 °C three times in 10 mM Tris, 5 mM EDTA, 1% phenylmethanesulphonylfluoride (PMSF), pH8.0, then incubated 37 °C, 24hrs in 0.25% Trypsin/0.1%EDTA; then washed in rinsing buffer (8 g/L NaCl, 200 mg/L KCl, 1 g/L Na2HPO4, 200 mg/L KH2PO4, 1% PMSF), 37° C, 20 min three times; then incubated 37° C, 24hrs in 55 mM Na2HPO4, 17 mM KH2PO4, 4.9 mM MgSO4*7H2O, 160 U/mL DNase I type II, 100 μg/mL RNase type IIIA, 80 U/mL lipase type VI-S (Sigma-Aldrich Inc., St. Louis MO, USA), 1% PMSF; then washed sequentially in rinsing buffer 37 °C, 20 min three times; 99.9% isopropanol, 1% PMSF 25 °C once for 24hrs; then washed in rinsing buffer 37 °C, 20 min three times; then washed in 70% EtOH, 37 °C, 20 min 3X three times; then washed in storage solution (PBS, 1% PMSF) 37 °C, 20 min once; then stored in storage solution 4 °C until use.

To prepare AGE-modified ECM, 200 mg VAT explants were cultured 72 hours, 37 °C in maintenance medium containing either 17 mM (Low glucose), 50 mM, or 100 mM (High glucose) glucose, or 100 mM mannitol, doses based on prior literature^59,66,67^ and dose-response experiments demonstrating that 100 mM glucose generated levels of AGE-modification similar to native DM adipose tissue (Fig. 3B). The deglycosylating enzyme PNGase-F, was added for the final 24 hours of 72-hour treatment (2.5 units/mL, Sigma-Aldrich Inc., St Louis, MO, USA, Cat#F8435-300UN). ECM was then prepared from treated tissues as described above.

### Scanning electron microscopy (SEM)

SEM was performed as described^6^. Briefly, tissues were fixed, mounted on SEM-stub with colloidal graphite, sputter-coated with gold, and images captured on an Amray 1910 scanning electron microscope.

### 2D adipocyte culture

2D-adipocyte culture was performed as described^6,68^. Briefly, adipose tissue was digested with Type II collagenase (2 mg/mL in PBS/2% BSA, Life Technologies Inc., Carlsbad, CA, USA) 37 °C, 60 min, centrifuged 250rcf, the stromal-vascular cell pellet retrieved, plated overnight, and adherent cells passaged 3X to enrich for preadipocytes, which were frozen in DMEM/F12, 15% fetal calf serum (FCS), 10% DMSO in liquid nitrogen until use. To generate mature adipocytes for 2D culture, preadipocytes (60,000 cells/well in 24-well plates) were plated in DMEM/F12, 15% FCS until confluent, cultured 7days in differentiation medium (DMEM/F12, 2.5 mM glutamine, 15 mM HEPES, 10 mg/ml transferrin, 33 μM biotin, 0.5 μM human insulin, 17 μM pantothenate, 0.1 μM dexamethasone, 2 nM T3, 540 μM IBMX, 1 μM ciglitazone), then cultured 7days in maintenance medium (DMEM/F12, 2.5 mM glutamine, 15 mM HEPES, 10 mg/ml transferrin, 33 μM biotin, 0.5 μM human insulin) until differentiated.

Unglycated or glycated recombinant human collagen 1 was added to culture media in soluble form for 2D culture. For glycation, recombinant human collagen 1 (Rockland Immunochemicals, Inc., Limerick, PA, USA, Cat#009-001-103) was incubated in 100 μg/ml with 500 mM D-glucose-6-phosphate in PBS, 4 weeks, 37 °C, and used at 20 μg/ml in culture, based on previously published methods^69^, as well as dose response studies we performed studying concentrations ranging from 20–100 μg/ml that demonstrated 20 μg/ml provided maximal effect on adipocyte glucose uptake.

### 3D adipocyte culture

3D-adipocyte culture was performed as described^6,68^. Decellularized human VAT ECM was rinsed in 70% EtOH, rehydrated in PBS, cut into 200 mg fragments, seeded with 60,000 preadipocytes in 20 μL of complete growth medium (DMEM, 10% FCS), incubated 37 °C, 5%CO_2_, 40 min to allow cells to adhere, then 0.5 mL of complete growth medium added, incubated 37 °C, 5%CO_2_, 24hrs, transferred to a fresh culture plate, 0.5 mL of complete growth medium added, cultured 3 days, then cultured in 0.5 mL differentiation medium 14 days to generate mature adipocytes in ECM.

RAGE neutralizing antibody (IgG2b mu-α-huRAGE) and IgG2b mu-α-hu isotype control antibody (Abcam Inc., Cambridge, MA, USA, Cat#ab89911, Cat#ab170192 respectively, 2 μg/mL) or CD36 neutralizing antibody (IgA mu-α-huCD36) and IgA mu-α-hu isotype control antibody (Abcam Inc., Cambridge, MA, USA, Cat#ab23680, Novus Biologicals, Centennial, CO, USA, Cat# NBP1-97030 respectively, 2 μg/mL) were added to ECM-adipocyte cultures throughout adipocyte differentiation and during glucose uptake assay; the hDia1 small molecule formin FH2-domain inhibitor SMIFH2^60,61^ (25 μM, Sigma-Aldrich Inc., St. Louis, MO, USA, Cat#344092) was used for 20 minutes prior to and throughout glucose uptake assay.

### Metabolic phenotyping

For glucose uptake assay, adipocytes in 2D-culture or 3D-ECM were cultured 37 °C, 72hrs in 0.5 mL maintenance medium; then in serum-free DMEM:F12, 37 °C, 12hrs; in PBS/1% BSA, 37 °C, 2hrs; washed in PBS, then cultured in 0.5 mL PBS +/−100 nM human insulin, 37 °C, 40 min; in 0.5 mL PBS +/− 200 nM insulin, 0.1 mM 2-deoxy glucose, 2 μCi/mL deoxy-D-glucose-2-[1,2-^3^ H(N)] (PerkinElmer Inc., Waltham, MA, USA) 37 °C, 40 min, then washed with PBS, and 420 μL 1% SDS solution added, and cells lysed with pipetting. 10 μL of cell lysate was used for Bradford protein assay; 400 μL of lysate was transferred to 2 mL scintillation fluid and counts per minute (cpm) measured on a scintillation counter and normalized to cell lysate protein concentration.

### RT-qPCR

Adipocytes were lysed in Trizol and RNA extracted with RNAEasy Fibrous Tissue MiniKit (Qiagen Inc., Hilden, Germany). Purity, concentration, and integrity of mRNA were evaluated using a NanoDrop 1000 spectrophotometer (Thermo Scientific, Wilmington, DE, USA). Equal amounts of input RNA were reverse-transcribed using the Applied Biosystems High Capacity cDNA Archive Kit (Applied Biosystems, Foster City, CA, USA). RT-qPCR was conducted with TaqMan primers and reagents (Life Technologies Inc., Carlsbad, CA, USA). Data are presented as fold changes calculated from least squares mean differences according to the 2^−ΔΔCt^ method^70^ and normalized to the mean of *B2M* and *GAPDH* housekeeping gene controls, for which Ct values did not change with GC1 treatment.

### Statistics

Statistical analysis was performed in STATA-version 15 (StataCorp LLC, College Station, TX, USA). Data was tested for normality and homoscedasticity and analyzed accordingly. The a-priori α-level was set at 5%. Means and standard errors of measures are displayed in figures. Linear mixed model was used to compare AGE-immunofluorescence in tissues by diabetes status, using an interaction term between diabetes status and depot to assess if differences between DM and NDM tissues were affected by depot. The relationship between AGE-immunofluorescence and HbA1c was estimated using linear mixed model in VAT and SAT separately (Fig. 1). In 2D-culture experiments, linear mixed model was used to detect differences in log-transformed glucose uptake by treatment (UC1, GC1) and diabetes status. An interaction term for treatment and diabetes status was used to assess if the effect of treatment on glucose uptake was different for DM and NDM patients. For RT-qPCR data, dCT values between experimental arms were compared using two-way ANOVA mixed model (Fig. 2). For 3D-culture experiments (Figs. 3, 4), linear mixed models were used to estimate the effect of different levels of ECM glycation (mannitol, low glucose, medium glucose, high glucose, PNGase as applicable), diabetes status, RAGE antibody, and SMIFH2, and interactions between these factors, on glucose uptake. Random intercept was used for all models to account for within-subject correlations, while controlling for age, sex, and BMI of both adipocyte and ECM patient source. Post-hoc pairwise comparisons were performed to detect differences in outcome variables at different levels of independent variables using Bonferroni’s correction for multiple testing. Figures present data as means with standard errors of mean.

## Supplementary information

Supplementary Figure 1

## Data Availability

All data generated and analyzed for this study are included in the published article and Supplementary Information Files. All reagents will be freely provided upon reasonable request, except for human tissue and cell samples, and human subject clinical information or identifying information, which are not permitted to be shared due to IRB, HIPAA, and confidentiality constraints.
