# Supplementary material for: Advanced glycation end-products regulate extracellular matrix-adipocyte metabolic crosstalk in diabetes
Source: Sci Rep. 2019 Dec 24;9:19748. doi: 10.1038/s41598-019-56242-z (PMC6930305; doi:10.1038/s41598-019-56242-z)
Supplement: Supplementary file 1 — Supplementary Figure 1 [file 41598_2019_56242_MOESM1_ESM.pdf]

SUPPLEMENTARY FIGURE 1:

Advanced glycation end-products regulate extracellular matrix-adipocyte metabolic crosstalk in diabetes

Clarissa Strieder-Barboza[1,2], Nicki A. Baker[1], Carmen G. Flesher[1], Monita Karmakar[1], Christopher K. Neeley[1], Dominic Polsinelli[5], Justin B. Dimick[1], Jonathan F. Finks[1], Amir A. Ghaferi[1,7], Oliver A. Varban[1], Carey N. Lumeng[2,3,4], Robert W. O'Rourke[1,6]*

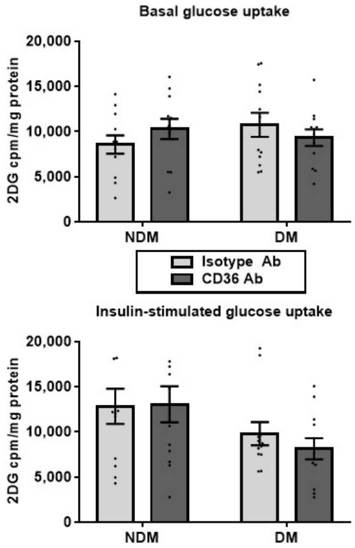

Supplementary Figure 1: CD36 does not regulate AGE-mediated ECM-adipocyte metabolic crosstalk in 3D-ECM culture. VAT preadipocytes differentiated *in vitro* into mature adipocytes +/- CD36 blocking antibody, or isotype control antibody in disease-matched (NDM or DM) VAT ECM treated with Low (17mM) glucose, then studied with glucose uptake assay without (basal) or with insulin stimulation (200 nM, 40 min). Ordinates: mean ^{3}H-2D-glucose uptake (cpm) normalized to cell lysate protein concentration (mg/ml); n= 16 NDM, 15 DM subjects.
